# The Routine Application of Tumor-Treating Fields in the Treatment of Glioblastoma WHO° IV

**DOI:** 10.3389/fneur.2022.900377

**Published:** 2022-06-16

**Authors:** Aleksandrs Krigers, Daniel Pinggera, Matthias Demetz, Lisa-Marie Kornberger, Johannes Kerschbaumer, Claudius Thomé, Christian F. Freyschlag

**Affiliations:** Department of Neurosurgery, Medical University of Innsbruck, Innsbruck, Austria

**Keywords:** glioblastoma, TTF = tumor-treating field, neurosurgery, neurooncology, combined treatment approach

## Abstract

**Introduction:**

Tumor-treating fields (TTFs) are a specific local oncological treatment modality in glioblastoma multiforme WHO° IV (GBM). Their mechanism of action is based on the effect of electrical fields interfering with the mitotic activity of malignant cells. Prospective studies have demonstrated efficacy, but TTF benefits are still controversially discussed. This treatment was implemented in our center as the standard of care in January 2016. We thus discuss the current state of the art and our long-term experience in the routine application of TTF.

**Methods:**

The data of 48 patients suffering from GBM and treated with TTF were assessed and compared with previously published studies. Up-to-date information from open sources was evaluated.

**Results:**

A total of 31 males and 17 females harboring a GBM were treated with TTF, between January 2016 and August 2021, in our center. In 98% of cases, TTFs were started within 6 weeks after concomitant radiochemotherapy (Stupp protocol). Mean overall survival was 22.6 months (95% CI: 17.3–27.9). Current indications, benefits, and restrictions were evaluated. Future TTF opportunities and ongoing studies were reviewed.

**Conclusion:**

TTFs are a feasible and routinely applicable specific oncological treatment option for glioblastoma multiforme WHO° IV. Further research is ongoing to extend the indications and the efficacy of TTF.

## Introduction

Glioma is the most frequent primary malignant tumor of the central nervous system (CNS) (1). High-grade glioma, especially glioblastoma multiforme WHO° IV (GBM), behaves aggressively with the corresponding unfavorable outcome and thus limited life expectancy (2). The established standardized treatment consists of a neurologically safe tumor resection (3–5), followed by adjuvant concomitant radiochemotherapy and six cycles of temozolomide (TMZ) monotherapy thereafter. This strategy is known as the Stupp protocol (6, 7). The disease, however, is considered incurable and there is a lack of efficient treatment options in the case of recurrent or progressive disease (8, 9).

Therefore, the demand for new approaches and targeted treatment options remains high. Although there is extensive research in this field, very few promising treatment options succeeded in the translation to clinical routine. One of them is the targeted application of tumor-treating fields (TTFs) (10). This method is based on the local effect of electric fields to interfere with the mitotic activity of the tumor. Proliferating cells are blocked in metaphase and anaphase as the formation of the mitotic spindle is disturbed, which results in slower cell replication or apoptosis (11–14).

TTFs are FDA- and EMA-approved for the treatment of adults with newly diagnosed GBM. In this case, TTFs are started within 6 weeks after the end of concomitant radiochemotherapy, ideally simultaneously with TMZ monotherapy (15, 16). Alternatively, TTF therapy can be an optional treatment in the case of recurrent GBM, overcoming the side effects of systemic second-line chemotherapeutics (17). Nevertheless, controversies considering TTF benefits are still conveyed (18).

Practically, four soft non-invasive adhesive electrode arrays are placed on the shaved head of the patient. The electric field is generated between the poles of the electrode arrays, which are supplied through a wire. The control device with pace-maker and the changeable accumulator is placed in a carry-on bag or backpack. Therapeutic success was seen when the device was worn for at least 18 h per day, with increasing benefits for every additional hour (15–17, 19). TTF therapy normally does not require an in-patient stay or additional oncological follow-ups. Technical assistance is provided by the service team of the manufacturer. Still, daily support by a person from the patient's household remains mandatory.

This kind of treatment in the case of GBM is incorporated in the international clinical guidelines (4, 20) and is implemented as a standard of care in our center since January 2016. During the following years, we gained practical know-how in TTF initiating, namely, informed consent, compliance, and follow-up. Thus, we aimed to discuss the current state of the art together with our long-term experience with the routine application of TTF. Moreover, we evaluated current indications, benefits, restrictions and also future TTF opportunities and ongoing studies.

## Materials and Methods

According to international guidelines (4, 20) and consequently internal standard operating procedures, all patients harboring a histologically proven new or recurrent glioblastoma WHO° IV since January 2016 were considered for TTF therapy. Neuropathological tumor assessment was performed in all cases according to the revised 4th WHO classification of CNS tumors (21). The TTF indication was individually confirmed by the multidisciplinary neuro-oncological tumor board. Regardless of TTF, elective clinical and MRI follow-ups were performed every 3 months, in which the general and neurological condition, compliance, potential side effects, and oncological status were checked. In the case of recurrent or progressive disease, the eligibility for TTF was discussed in the multidisciplinary neuro-oncological tumor board. If there were no beneficial options for oncological resection, targeted systemic, or radiotherapy, TTF could be offered in case of expected compliance. TTF therapy is accepted by the Austrian healthcare insurance and, after formal approval of the indication, treatment costs are covered.

We assessed all patients who received the entire neuro-oncological treatment (surgery, radiation therapy, and chemotherapy) from the TTF starting in January 2016 till August 2021 from the institutional database. Patients who received either one of recommended treatments in external institutions were excluded. Each case data, namely, epidemiological, clinical, neuropathological, and follow-up records, was collected in the institutional database. The evaluation of this information for the scientific purpose was approved by the ethics committee of the Medical University of Innsbruck (No.: 1402/2020). It was performed in accordance with the ethical standards as laid down in the 1964 Declaration of Helsinki and its later amendments or comparable ethical standards.

All available publications and open-source data considering TTF application in high-grade gliomas were gathered and evaluated.

## Results

A total of 48 patients harboring a GBM treated with TTF were evaluated. Surgical intervention was performed in all cases, whereas gross neurologically safe resection was performed in 44 (92%) and biopsy in 4 (8%) patients. Among the patients, which were treated with TTF in our center, 31 (65%) were men and 17 (35%) were women with a median age of 57 years (interquartile range (IqR): 44–62), whereas 18 (38%) patients were older than 60 years and one was 16. The isocitrate dehydrogenase (IDH) status was routinely evaluated in 38 cases: IDH was mutated in 9 (23%) and remained wild-type in 30 (67%) tumors. In 22 (60%) patients, O(6)-methylguanine-DNA methyltransferase (MGMT) promoter was methylated and in 15 (40%) it stayed unmethylated. The Karnofsky performance index before surgery amounted to 80–100. Epileptic seizures before the surgery were reported in 13 patients. During TTF treatment, epileptic seizures were found in 6 patients, whereas in 2 cases a patient had them before the surgery and in 4 cases epilepsy developed *de novo* after it. TTFs were administered for the recurrent GBM in a single case only.

If the eligible patient agreed with the initiation of TTF, the service team arranged an appointment, which was usually held in our outpatient department. During this meeting, the technical and everyday aspects were discussed, and the patient and the assistant were trained to operate the device. The service team is available for technical questions around the clock. It was essential, that a person from the patient's household was ready to help in daily activities considering device management. As it is crucial, that the system is active for at least 75% of the time, the automatically generated compliance reports were sent to our center monthly. At the same time, no report concerning the interaction between a patient and the service team was usually available. Of course, any unplanned or urgent visit to our center remained possible.

The daily support was mostly provided by a partner (15/25, 60%), children (5/25, 20%), parents (3/25, 12%), siblings (1/25, 4%), or friends (1/25, 4%). No defined assistant was specified by 23 patients.

No TTF-associated serious adverse effects were reported during the median follow-up of 17 months (IqR: 11–23). No interruption of follow-ups or technical service, hence, TTF application, was noticed due to the COVID-19 pandemic, the associated lock-down, or due to limited access to healthcare facilities.

Mean overall survival inside our cohort according to Kaplan–Meier assessment was 22.6 months (95% CI: 17.3–27.9). IDH-status did not predict OS among patients with TTF therapy, which remained 25.7 months (95% CI: 14.0–37.4) in case of IDH mutation or 20.8 months (95% CI: 15.6–26.1) in case of IDH wild-type tumor, LogRank *p* = 0.549. At the same time, OS was significantly more favorable in the case of methylated MGMT promoter: 29.7 months (95% CI: 22.4–37.0) vs. 16.7 months (95% CI: 11.4–22.0), LogRank *p* = 0.002.

## Discussion

### Current Status

Tumor-treating fields are an established option in case of recurrent or newly diagnosed GBM. Their practical and routine application is feasible, nevertheless, some points require attention.

In the PubMed database, 274 results were associated with the term “Tumor-Treating Fields,” showing a strong rise in recent years: 15 papers were published in 2015, 39 papers in 2019, and already 57 in 2021. Nevertheless, more than 80% of them are reviews, preclinical studies or are attributed to non-CNS tumors. Thus, even if TTF as a specific oncological option is widely discussed, the translational and applied experience remains limited.

Tumor-treating fields are the first treatment option in many years, that has shown successful results in GBM therapy (10). It is labeled as the fourth modality in cancer therapy among surgery, systemic pharmacological, and radiation therapy (22).

The EF-11 study from 2012 was performed on 237 patients (1:1 randomization). Even if it did not demonstrate the superiority of TTF compared to the local standard of care in the case of recurrent GBM, the safety and routine feasibility of TTF were proven (17). The pivotal EF-14 trial on 695 patients (2:1 randomization), which was published in 2015 and consistently actualized in 2017, showed a statistically significant overall survival benefit of 4.9 months for patients harboring newly diagnosed GBM. Moreover, 2- and 5-year survival was significantly more favorable in the TTF group compared to the control arm as well – 43 vs. 31% (*p* < 0.001) and 13 vs. 5% (*p* = 0.004), respectively (15, 16, 23). According to both studies, the TTF was approved for newly diagnosed and recurrent GBM by FDA and EMA and consequently included in the guidelines (4, 20). Our survival data are concordant to EF-14 published material with comparable overall survival of 22.6 vs. 20.9 months, whereas selection bias in the case of clinical routine could play a role. According to our data, MGMT promoter methylation provides significant additional survival benefits even within the TTF cohort.

In our center, TTF was only applied to a single patient with recurrent GBM. It is known that a prolonged period of time is necessary until TTF effects can be observed (24). In the case of recurrent GBM, the length of therapy remains short and usually consists only of several months, as was shown in the EF-11 and PriDE studies (17, 25).

There are practical advantages of TTF compared to other specific oncological treatment modalities with the noninvasiveness being a key point. Moreover, during hygienic procedures, sports, and MRI the device can be put off. More than one-third of the patient in our cohort were older than 60 years. The feasibility and safety of an elderly population were also shown in the subgroup analysis of the EF-14 study (26). Hence, TTFs are also feasible in the case of an aged population, even when the full dose of radiochemotherapy is not suggested. Another point is that TTF localized treatment allows it to be considered in settings where conventional cytotoxic chemotherapy may be contraindicated due to systemic complications and/or adverse events.

No clinically relevant TTF interaction with the radiotherapy field was found (27). Moreover, TTFs delay DNA damage repair following conventional photon-beam radiotherapy (28) and work as a sensitizer for proton-beam therapy (29). According to the literature, dexamethasone administration does also not interfere with TTF (30).

There were concerns that the COVID-19 pandemic could interfere with the TTF support (31). In our center, however, no interruption of the TTF service and follow-ups were observed.

Even if TTFs are permitted by FDA and EMA, their limitations and potential drawbacks remain intensely discussed. The approval EF-11 and EF-14 studies were open-labeled, thus, providing potential bias. On the other hand, there was no technical possibility to randomize by imitation of the working device, as the arrays cause a superficial warmth sensation. Another potential drawback is the lack of industry-independent validation trials, which, however, is the case for most (pharmaceutical) oncological treatment studies as well. In addition, EF-11 was criticized due to the heterogeneous previous treatment and control population.

We did not observe any severe adverse effects in the routine application of the TTF system. Only skin irritation was reported as a device-related side effect in the EF-11 and EF-14 studies. In 2% of cases, severe local skin damage was described. In the EF-14 trial for newly diagnosed GBM patients, where the treatment exposure was longer than that for recurrent disease, grades 1 and 2 skin reactions were reported in 43% of patients. Similar data were shown during a phase 4 study with 11.000 patients (32). Therefore, sensitivity to the conductive hydrogel, which is used as an adhesive agent for the electrodes, is mentioned as a limitation for starting the therapy. Recently, the prediction algorithm for skin irritation probability was presented. According to it, the variation of array positioning reduced the risk of skin irritation by about one-third (33). Moreover, practical suggestions for dermatological symptomatic treatment have been distributed (34).

We have mentioned that the daily help in routine maintenance of the device from the side of relatives or another assisting person is needed. Every 3-day shaving, application of adhesive electrodes, technical device management, and even contact with the service team is time-consuming. The favorable effect of TTF is magnified if the device is active for more than 18 h per day based on the *post-hoc* subgroup analysis of the EF-14 trial. Moreover, the survival benefit rises with each added hour (19). Thus, even if a patient showed a high-performance index like KPI 80–100 as in our series, external support remains essential. On the other hand, even if the daily life of patients might be affected by the TTF, two-thirds of them would decide in favor of the treatment (35). The quality of life and TTF acceptance remained high (36, 37). Moreover, the technical upgrade of the device like reduced weight and higher accumulator capacity solved some problems (38).

Another point is the limited data in the case of an implanted CSF shunt or pacemaker that can potentially interfere with TTF. Nevertheless, case reports and a retrospective study demonstrated a high likelihood of to use of TTF devices even in these patients (39, 40), but larger trials would be necessary.

The cost-effectiveness was discussed, as monthly costs of about 20.000*e* are to be considered (35, 41, 42) and the high price was thought to limit the access to this treatment option (41). Nevertheless, the insurances in the United States and several European countries do cover the routine application of TTF, preventing costs for patients.

Thus, the TTF therapy is nowadays advisable for all patients harboring GBM, including frailty ones, when demanding high-dose radiochemotherapy could not be applied. The efficacy of dexamethasone or radiotherapy is not negatively interfered with. Nevertheless, the external support from relatives and compliance stays crucial. There are also other restrictions like cranial or active implants. Skin irritation does not look to be an inevitable restriction. The summary of positive and negative points considering the TTF application is provided in Table 1.

**Table 1:** Applied advances and limitations of TTF therapy nowadays.

**Advances**	**Limitations**
A novel effective therapy option for GBM	Translational experience is limited
Approval in guidelines	Criticism of pivotal trials
Non-invasiveness	Requires skin preparation and shaving
Possible in aged / frailty patients	Several implants remain contraindication
TTF acceptance is good	Device requires extra bag
High quality of life	Active use at least 18 h a day
MGMT promoter status remains relevant	Support from family remains important
Skin irritation is rarely severe	Skin irritation comes in 2% of cases
Costs are covered in some	High device expenses
EU countries	
Possible sensitizer for radiotherapy	
Does not interfere with dexamethasone	

### Future Clinical Opportunities

Research regarding further applications of TTF is ongoing. The TRIDENT (EF-32, NCT04471844) study was recently initiated to demonstrate the potential benefits of TTF starting parallel to concomitant radiochemotherapy in the case of newly diagnosed GBM. The feasibility and safety were checked on 10 patients during a phase 1 study (43). The results of an analogous 1:1 randomized phase 2 study (NCT03869242) on 60 patients with an estimated completion date is December 2021 have not been published yet.

The combined treatment option of TTF and bevacizumab (BEV) in the case of recurrent GBM was suggested already in 2014 (44). Nevertheless, the study evaluating concomitant TTF with BEV and hypofractionated stereotactic radiotherapy was abandoned due to poor recruiting in 2019 (NCT01925573). On the other hand, there are reports that BEV with concomitant TTF is applied in an off-label fashion. In one retrospective study, all 48 patients received BEV as monotherapy or in combination with other systemic chemotherapeutics concomitant to TTF. However, due to the lack of a control arm, which would include BEV cases without TTF therapy, no conclusion regarding a potential co-influence of TTF and BEV is possible (45).

According to the guidelines, the treatment strategy in the case of anaplastic glioma is often similar to glioblastoma (4, 20). Nevertheless, the data considering TTF application for anaplastic glioma are limited. Due to the high clinical demand, a respective study is currently ongoing (46).

No sufficient data are available for pediatric cases. According to a case series of 4 patients under 16 (2 of them with GBM) 53–92% compliance without severe adverse effects was described (47). In another publication, the partial response was shown in 5 pediatric high-grade glioma cases (48). Large trials on a pediatric population are ongoing (49).

There is a single spinal glioma case, in which TTF was applied for primary thoracolumbar anaplastic astrocytoma. After decompression surgery and adjuvant chemoradiotherapy, one adhesive array was placed above the tumor projection on the back and another below it. According to the virtual modeling, this way of application provided sufficient TTF power density at the target site (50).

An enhancing effect of TTF after skull remodeling surgery was described. For superficial tumors, removal of a standard craniotomy bone flap increased the electrical field strength by up to 70% (51). A phase 1 safety study on 15 patients with recurrent GBM confirmed the safety of this approach (52) and a phase 2 study was announced (53).

The exact molecular pathways of the TTF effect remain unclear (54). Multiple studies here are ongoing. On the other hand, there is encouraging preclinical data considering increased synergistic efficacy of checkpoint inhibitor anti-PD-1 therapy when combined with TTF. It was demonstrated that the volume of two tumor models declined and the number of cancer-infiltration immune cells raised when TTF was added to the anti-PD-1 therapy (55). Similar results were shown in another study of non-small cell lung cancer (NSCLC), where the concomitant TTF and checkpoint inhibitor treatment led to a decrease in the tumor volume (56).

Tumor-treating field (TTF) application is being investigated also for other solid cancers: the LUNAR trial for lung cancer, HEPANOVA for hepatocellular cancer, INNOVATE-3 for ovarian cancer, PANOVA-3 for pancreatic cancer, and METIS for brain metastasis (57). According to the results of the STELLAR study, a specific device modification is approved by FDA for adult patients harboring unresectable, advanced, and malignant pleural mesothelioma together with standard chemotherapy (58).

## Conclusion

Tumor-treating fields are a feasible and routinely applicable specific oncological treatment option in the case of glioblastoma multiforme WHO° IV. As TTF provides additional overall survival, this option should be presented and advised to all GBM patients. Nevertheless, practical restrictions stay relevant, which limit the usage of this modality like insufficient external assistance. Additional work is necessary and is intensely ongoing to extend the indications and the efficacy of TTF and to reduce restrictions.

## Data Availability Statement

The raw data supporting the conclusions of this article will be made available by the authors, without undue reservation.

## Ethics Statement

The studies involving human participants were reviewed and approved by Ethics Committee of the Medical University of Innsbruck. Written informed consent from the patients/participants or patients/participants' legal guardian/next of kin was not required to participate in this study in accordance with the national legislation and the institutional requirements.

## Author Contributions

AK: acquisition, analysis of data, interpretation of data, and drafting the article. JK, DP, MD, and L-MK: acquisition and interpretation of data. CFF and CT: design of the study and revisions. CFF: conception/design of the study and interpretation of data. All authors have approved the submitted version and have agreed with both to be personally accountable for the author's own contributions and to ensure that questions related to the accuracy or integrity of any part of the work, even ones in which the author was not personally involved, have been appropriately investigated, resolved, and the resolution documented in the literature.

## Conflict of Interest

CFF declares he is participating in a speaker bureau and an advisory board of Novocure. The remaining authors declare that the research was conducted in the absence of any commercial or financial relationships that could be construed as a potential conflict of interest.

## Publisher's Note

All claims expressed in this article are solely those of the authors and do not necessarily represent those of their affiliated organizations, or those of the publisher, the editors and the reviewers. Any product that may be evaluated in this article, or claim that may be made by its manufacturer, is not guaranteed or endorsed by the publisher.

## References

[1] Ostrom QT Patil N Cioffi G Waite K Kruchko C Barnholtz-Sloan JS. CBTRUS statistical report: primary brain and other central nervous system tumors diagnosed in the United States in 2013-2017. Neuro Oncol. (2020) 22:iv1–1v96. 10.1093/neuonc/noaa200 33123732 PMC7596247

[2] Poon MTC Sudlow CLM Figueroa JD Brennan PM. Longer-term (>/= 2 years) survival in patients with glioblastoma in population-based studies pre- and post-2005: a systematic review and meta-analysis. Sci Rep. (2020) 10:11622. 10.1038/s41598-020-68011-4 32669604 PMC7363854

[3] Weller M van den Bent M Hopkins K Tonn JC Stupp R Falini A. European Association for Neuro-Oncology Task Force on Malignant, EANO guideline for the diagnosis and treatment of anaplastic gliomas and glioblastoma. Lancet Oncol. (2014) 15:e39515:4t 10.1016/S1470-2045(14)70011-7 25079102

[4] Wen PY Weller M Lee EQ Alexander BM Barnholtz-Sloan JS Barthel FP. Glioblastoma in adults: a Society for Neuro-Oncology (SNO) and European society of neuro-oncology (EANO) consensus review on current management and future directions. Neuro Oncol. (2020) 22:1073–073: 10.1093/neuonc/noaa106 32328653 PMC7594557

[5] Stupp R Brada M Van Den Bent MJ Tonn JC Pentheroudakis GE. High-grade glioma: ESMO Clinical Practice Guidelines for diagnosis, treatment and follow-up. Ann Oncol. (2014) 25(Suppl 3):iii93–101. 10.1093/annonc/mdu050 24782454

[6] Stupp R Hegi ME Mason WP Van Den Bent MJ Taphoorn MJ Janzer RC. Effects of radiotherapy with concomitant and adjuvant temozolomide vs. radiotherapy alone on survival in glioblastoma in a randomised phase III study: 5-year analysis of the EORTC-NCIC trial. Lancet Oncol. (2009) 10 459–66. 10.1016/S1470-2045(09)70025-7 19269895

[7] Stupp R Mason WP van den Bent MJ Weller M Fisher B Taphoorn MJ. National Cancer Institute of Canada Clinical Trials, Radiotherapy plus concomitant and adjuvant temozolomide for glioblastoma N Engl J Med. (2005) 352:987–96. 10.1056/NEJMoa043330 15758009

[8] Weller M Cloughesy T Perry JR Wick W. Standards of care for treatment of recurrent glioblastoma–are we there yet? Neuro Oncol. (2013) 15:4Onco 10.1093/neuonc/nos273 23136223 PMC3534423

[9] Chen W Wang Y Zhao B Liu P Liu L Wang Y. Optimal therapies for recurrent glioblastoma: a bayesian network meta-analysis. Front Oncol. (2021) 11:641878. 10.3389/fonc.2021.641878 33854975 PMC8039381

[10] M. Penas-Prado. Practice-changing abstracts from the 2016 society for neuro-oncology annual scientific meeting. Am Soc Clin Oncol Educ Book. (2017) 37:187kk B 10.1200/EDBK_175563 28561706

[11] Carrieri FA Smack C Siddiqui I Kleinberg LR Tran PT. Tumor treating fields: at the crossroads between physics and biology for cancer treatment. Front Oncol. (2020) 10:575992. 10.3389/fonc.2020.575992 33215030 PMC7664989

[12] Wenger C Giladi M Bomzon Z Salvador R Basser PJ Miranda PC. Modeling Tumor Treating Fields (TTFields) application in single cells during metaphase and telophase. Annu Int Conf IEEE Eng Med Biol Soc. (2015) 2015:6892–89 10.1109/EMBC.2015.7319977 26737877

[13] Silginer M Weller M Stupp R Roth P. Biological activity of tumor-treating fields in preclinical glioma models. Cell Death Dis. (2017) 8:e2753. 10.1038/cddis.2017.171 28425987 PMC5477589

[14] Gera N Yang A Holtzman TS Lee SX Wong ET Swanson KD. Tumor treating fields perturb the localization of septins and cause aberrant mitotic exit. PLoS ONE. (2015) 10:e0125269. 10.1371/journal.pone.0125269 26010837 PMC4444126

[15] Stupp R Taillibert S Kanner A Read W Steinberg D Lhermitte B. Effect of tumor-treating fields plus maintenance temozolomide vs. maintenance temozolomide alone on survival in patients with glioblastoma: a randomized clinical trial. JAMA. (2017) 318:2306–306 10.1001/jama.2017.18718 29260225 PMC5820703

[16] Stupp R Taillibert S Kanner AA Kesari S Steinberg DM Toms SA. Maintenance therapy with tumor-treating fields plus temozolomide vs. temozolomide alone for glioblastoma: a randomized clinical trial. JAMA. (2015) 314:2535–535 10.1001/jama.2015.16669 26670971

[17] Stupp R Wong ET Kanner AA Steinberg D Engelhard H Heidecke V. NovoTTF-100A vs. physician's choice chemotherapy in recurrent glioblastoma: a randomised phase III trial of a novel treatment modality. Eur J Cancer. (2012) 48:2192–192: 10.1016/j.ejca.2012.04.011 22608262

[18] Lassman AB Joanta-Gomez AE Pan PC Wick W. Current usage of tumor treating fields for glioblastoma. Neurooncol Adv. (2020) 2: vdaa069. 10.1093/noajnl/vdaa069 32666048 PMC7345837

[19] Toms SA Kim CY Nicholas G Ram Z. Increased compliance with tumor treating fields therapy is prognostic for improved survival in the treatment of glioblastoma: a subgroup analysis of the EF-14 phase III trial. J Neurooncol. (2019) 141:467lolp 10.1007/s11060-018-03057-z 30506499 PMC6342854

[20] Nabors LB Portnow J Ahluwalia M Baehring J Brem H Brem S. Central nervous system cancers, version 3.2020, NCCN clinical practice guidelines in oncology. J Natl Compr Canc Netw. (2020) 18:1537–70. 10.6004/jnccn.2020.0052 33152694

[21] Louis DN Perry A Reifenberger G von Deimling A Figarella-Branger D Cavenee WK. The 2016 World health organization classification of tumors of the central nervous system: a summary. Acta Neuropathol. (2016) 131:803ollt 10.1007/s00401-016-1545-1 27157931

[22] Mun EJ Babiker HM Weinberg U Kirson ED Von Hoff DD. Tumor-treating fields: a fourth modality in cancer treatment. Clin Cancer Res. (2018) 24:266Res 10.1158/1078-0432.CCR-17-1117 28765323

[23] Taphoorn MJB Dirven L Kanner AA Lavy-Shahaf G Weinberg U Taillibert S. Influence of treatment with tumor-treating fields on health-related quality of life of patients with newly diagnosed glioblastoma: a secondary analysis of a randomized clinical trial. JAMA Oncol. (2018) 4:495colis 10.1001/jamaoncol.2017.5082 29392280 PMC5885193

[24] Zhu P Zhu JJ. Tumor treating fields: a novel and effective therapy for glioblastoma: mechanism, efficacy, safety and future perspectives. Chin Clin Oncol. (2017) 6:41. 10.21037/cco.2017.06.29 28841803

[25] Mrugala MM Engelhard HH Dinh Tran D Kew Y Cavaliere R Villano JL. Clinical practice experience with NovoTTF-100A system for glioblastoma: the patient registry dataset (PRiDe). Semin Oncol. (2014) 41 Suppl 6:S4–S13. 10.1053/j.seminoncol.2014.09.010 25213869

[26] Ram Z Kim CY Hottinger AF Idbaih A Nicholas G Zhu JJ. Efficacy and safety of tumor treating fields (TTFields) in elderly patients with newly diagnosed glioblastoma: subgroup analysis of the Phase 3 EF-14 clinical trial. Front Oncol. (2021) 11:671972. 10.3389/fonc.2021.671972 34692470 PMC8526342

[27] Straube C Oechsner M Kampfer S Scharl S Schmidt-Graf F Wilkens JJ. Dosimetric impact of tumor treating field (TTField) transducer arrays onto treatment plans for glioblastomas - a planning study. Radiat Oncol. (2018) 13:31. 10.1186/s13014-018-0976-3 29471879 PMC5824562

[28] Giladi M Munster M Schneiderman RS Voloshin T Porat Y Blat R. Tumor treating fields (TTFields) delay DNA damage repair following radiation treatment of glioma cells. Radiat Oncol. (2017) 12:206. 10.1186/s13014-017-0941-6 29284495 PMC5747183

[29] Lee WS Seo SJ Chung HK Park JW Kim JK Kim EH. Tumor-treating fields as a proton beam-sensitizer for glioblastoma therapy. Am J Cancer Res. (2021) 11:4582–94. 34659907 PMC8493382

[30] Linder B Schiesl A Voss M Rodel F Hehlgans S Gullulu O. Dexamethasone treatment limits efficacy of radiation, but does not interfere with glioma cell death induced by tumor treating fields. Front Oncol. (2021) 11:715031. 10.3389/fonc.2021.715031 34395289 PMC8361446

[31] Gatson NTN Barnholtz-Sloan J Drappatz J Henriksson R Hottinger AF Hinoul P. Tumor treating fields for glioblastoma therapy during the COVID-19 pandemic. Front Oncol. (2021) 11:679702. 10.3389/fonc.2021.679702 34026655 PMC8139188

[32] Shi W Blumenthal DT Oberheim Bush NA Kebir S Lukas RV Muragaki Y. Global post-marketing safety surveillance of Tumor Treating Fields (TTFields) in patients with high-grade glioma in clinical practice. J Neurooncol. (2020) 148:489lols 10.1007/s11060-020-03540-6 32535723 PMC7438370

[33] Nour Y Pottgen C Kebir S Lazaridis L Ludemann L Guberina M. Dosimetric impact of the positioning variation of tumor treating field electrodes in the PriCoTTF-phase I/II trial. J Appl Clin Med Phys. (2021) 22:242sPhy 10.1002/acm2.13144 33389825 PMC7856507

[34] Lacouture ME Anadkat MJ Ballo MT Iwamoto F Jeyapalan SA La Rocca RV M. Prevention and management of dermatologic adverse events associated with tumor treating fields in patients with glioblastoma. Front Oncol. (2020) 10:1045. 10.3389/fonc.2020.01045 32850308 PMC7399624

[35] Onken J Goerling U Heinrich M Pleissner S Krex D Vajkoczy P. Patient reported outcome (PRO) among high-grade glioma patients receiving TTFields treatment: a two center observational study. Front Neurol. (2019) 10:1026. 10.3389/fneur.2019.01026 31681134 PMC6797850

[36] Zhu JJ Demireva P Kanner AA Pannullo S Mehdorn M Avgeropoulos N. Health-related quality of life, cognitive screening, and functional status in a randomized phase III trial (EF-14) of tumor treating fields with temozolomide compared to temozolomide alone in newly diagnosed glioblastoma. J Neurooncol. (2017) 135:545lole 10.1007/s11060-017-2601-y 28849310 PMC5700237

[37] Onken J Staub-Bartelt F Vajkoczy P Misch M. Acceptance and compliance of TTFields treatment among high grade glioma patients. J Neurooncol. (2018) 139:177lolm 10.1007/s11060-018-2858-9 29644485

[38] Kinzel A Ambrogi M Varshaver M Kirson ED. Tumor treating fields for glioblastoma treatment: patient satisfaction and compliance with the second-generation optune((R)) system. Clin Med Insights Oncol. (2019) 13:1179554918825449. 10.1177/1179554918825449 30728735 PMC6351720

[39] Kew Y Oberheim NA. Safety profile of tumor treating fields in adult glioblastoma patients with implanted non-programmable shunts, programmable shunts, and pacemakers/defibrillators: 6-year updated retrospective analysis. Int J Radiation Oncol^*^Biol^*^Physics. (2018) 102:e269102. 10.1016/j.ijrobp.2018.07.873

[40] McClelland III S Henrikson CA Ciporen JN Jaboin JJ Mitin T. Tumor treating fields utilization in a glioblastoma patient with a preexisting cardiac pacemaker: the first reported case. World Neurosurg. (2018) 119:58–60. 10.1016/j.wneu.2018.07.162 30064027

[41] Connock M Auguste P Dussart C Guyotat J Armoiry X. Cost-effectiveness of tumor-treating fields added to maintenance temozolomide in patients with glioblastoma: an updated evaluation using a partitioned survival model. J Neurooncol. (2019) 143:605lole 10.1007/s11060-019-03197-w 31127507

[42] Bernard-Arnoux F Lamure M Ducray F Aulagner G Honnorat J Armoiry X. The cost-effectiveness of tumor-treating fields therapy in patients with newly diagnosed glioblastoma. Neuro Oncol. (2016) 18:1129–129 10.1093/neuonc/now102 27177573 PMC4933490

[43] Bokstein F Blumenthal D Limon D Harosh CB Ram Z Grossman R. Concurrent tumor treating fields (TTFields) and radiation therapy for newly diagnosed glioblastoma: a prospective safety and feasibility study. Front Oncol. (2020) 10:411. 10.3389/fonc.2020.00411 32373508 PMC7186440

[44] Omar AI. Tumor treating field therapy in combination with bevacizumab for the treatment of recurrent glioblastoma. J Vis Exp. (2014) 27:e51638. 10.3791/51638 25407354 PMC4541554

[45] Lu G Rao M Zhu P Liang B El-Nazer RT Fonkem E. Triple-drug therapy with bevacizumab, irinotecan, and temozolomide plus tumor treating fields for recurrent glioblastoma: a retrospective study. Front Neurol. (2019) 10:42. 10.3389/fneur.2019.00042 30766509 PMC6366009

[46] O004290. ctive sacizumab, irinoT, Fu B, Bota D. Actr-41 A phase Ii, Single Arm study of optune® in bevacizumab-naive subjects with recurrent who grade Iii Malignant glioma. Neuro-Oncology. (2016) 18:vi1111i11. 10.1093/neuonc/now212.039

[47] Crawford J Saria MG Dhall G Margol A Kesari S. Feasibility of treating high grade gliomas in children with tumor-treating fields: a case series. Cureus. (2020) 12:e10804. 10.7759/cureus.10804 33163308 PMC7641478

[48] Green AL Mulcahy Levy JM Vibhakar R Hemenway M Madden J Foreman N. Tumor treating fields in pediatric high-grade glioma. Childs Nerv Syst. (2017) 33:1043–04 10.1007/s00381-017-3431-0 28470383

[49] Makimoto A Nishikawa R Terashima K Kurihara J Fujisaki H Ihara S. Tumor-treating fields therapy for pediatric brain tumors. Neurol Int. (2021) 13:151ntie 10.3390/neurolint13020015 33917660 PMC8167650

[50] De Los Santos J Arvatz S Zeevi O. Innv-05. Tumor treating fields (Ttfields) treatment planning for a patient with astrocytoma in the spinal cord. Neuro-Oncol. (2020) 22:ii117-ii117. 10.1093/neuonc/noaa215.489

[51] Korshoej AR Saturnino GB Rasmussen LK von Oettingen G Sorensen JC Thielscher A. Enhancing predicted efficacy of tumor treating fields therapy of glioblastoma using targeted surgical craniectomy: a computer modeling study. PLoS One. (2016) 11:e0164051. 10.1371/journal.pone.0164051 27695068 PMC5047456

[52] Korshoej AR Lukacova S Lassen-Ramshad Y Rahbek C Severinsen KE Guldberg TL. OptimalTTF-1: Enhancing tumor treating fields therapy with skull remodeling surgery. A clinical phase I trial in adult recurrent glioblastoma. Neurooncol Adv. (2020) 2:vdaa121. 10.1093/noajnl/vdaa121 33215088 PMC7660275

[53] Mikic N Poulsen FR Kristoffersen KB Laursen RJ Guldberg TL Skjøth-Rasmussen J. Study protocol for OptimalTTF-2: enhancing tumor treating fields with skull remodeling surgery for first recurrence glioblastoma: a phase 2, multi-center, randomized, prospective, interventional trial. BMC Cancer. (2021) 21:1010. 10.1186/s12885-021-08709-4 34503460 PMC8427888

[54] Hong P Kudulaiti N Wu S Nie J Zhuang D. Tumor treating fields: a comprehensive overview of the underlying molecular mechanism. Expert Rev Mol Diag. (2021) 22:19–28. 10.1080/14737159.2022.2017283 34883030

[55] Voloshin T Kaynan N Davidi S Porat Y Shteingauz A Schneiderman RS. Tumor-treating fields (TTFields) induce immunogenic cell death resulting in enhanced antitumor efficacy when combined with anti-PD-1 therapy. Cancer Immunol Immunother. (2020) 69:1191–191: 10.1007/s00262-020-02534-7 32144446 PMC7303058

[56] Weinberg U Voloshin T Yitzaki OT Kaynan N Giladi M Shteingauz A. Efficacy of Tumor Treating Fields (TTFields) and anti-PD-1 in non-small cell lung cancer (NSCLC) preclinical models. Annals Oncol. (2017) 28:ii11128 10.1093/annonc/mdx089.010

[57] Available, online at: www.novocure.com, accessed online on 11.12.2021

[58] Ceresoli GL Aerts JG Dziadziuszko R Ramlau R Cedres S van Meerbeeck JP. Tumor Treating Fields in combination with pemetrexed and cisplatin or carboplatin as first-line treatment for unresectable malignant pleural mesothelioma (STELLAR): a multicentre, single-arm phase 2 trial. Lancet Oncol. (2019) 20:1702–70 10.1016/S1470-2045(19)30532-7 31628016

